# Tumor targetable and pH-sensitive polymer nanoparticles for simultaneously improve the Type 2 Diabetes Mellitus and malignant breast cancer

**DOI:** 10.1080/21655979.2022.2060721

**Published:** 2022-04-12

**Authors:** Shi Tang, Peiqi Wen, Kaiheng Li, Jiehua Deng, Bo Yang

**Affiliations:** Department of Breast Surgery, Dongguan City Maternal & Children Health Hospital, Dongguan, Guangdong, China

**Keywords:** Type 2 Diabetes Mellitus, malignant breast cancer, tumor targeting, pH-sensitive, nanoparticles

## Abstract

In the recent study, we have developed novel tumor targetable and pH-sensitive PLGA nanoparticles co-loaded with camptothecin (CPT) and metformin (Metf) to simultaneously improve the Type 2 Diabetes Mellitus (T2DM) and malignant breast cancer. To improve the drug loading efficiency, the hydrophobic CPT was conjugated with PLGA polymer by the pH-sensitive hydrazone bonds (hyd). Then, the Metf was physically loaded into the hydrophobicity inner core of CPT-conjugated PLGA nanocomplex to form the dual drugs-loaded nanoparticles (NP/CPT-Metf). Furthermore, on the surface of NP/CPT-Metf was modified with tumor-homing CGKRK peptides to obtain the tumor targetable and pH-sensitive polymer nanoparticles (CNP/CPT-Metf). It was demonstrated that the developed CNP/CPT-Metf displayed sufficient sensitivity to the weak acidic tumor microenvironment. Besides, excellent ability of CNP/CPT-Metf to mediate accumulation of drugs in cells and tumor tissues finally in turn resulted in a signal enhanced anti-tumor effect. Furthermore, it was demonstrated as well that CNP/CPT-Metf was able of significantly alleviating the type 2 diabetes mellitus in diabetic mice. In summary, the developed multifunctional polymer nanoparticles might represent a promising strategy for simultaneously improve the T2DM and treat malignant breast cancer.

## GRAPHICAL ABSTRACT

**Figure:**
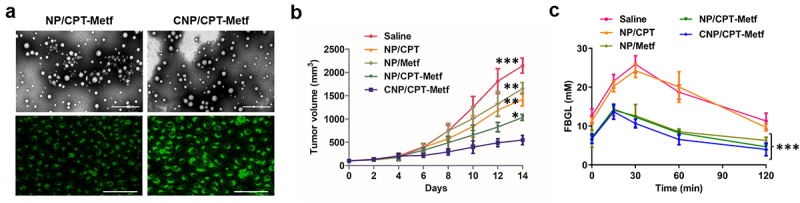

## Introduction

According to the data published by the Centers for Disease Control and Prevention, malignant breast cancer and diabetes are the leading causes of death among women with an estimated 268,600 new cases in a year [1]. Accumulating evidences demonstrated that a greater risk of developing breast cancer was found in those patients with type 2 diabetes [2–4]. It was demonstrated that women with diabetes have a 23% higher risk for developing breast cancer than those without diabetes [5]. Moreover, previous studies have found that preexisting diagnosis of diabetes was closely related to a 37% risk for all-cause mortality among women with breast cancer [6,7]. Additionally, there was certain similar symptoms between the diabetes patients and breast cancer patients and the symptoms of breast cancer could be exacerbated by diabetes [6,8].

Metformin (Metf) is a well-known biguanide and recommended as the first-line treatment of T2DM for decades [9]. As one of the most widely prescribed antihyperglycemic drugs, Metf has been noted in several international guidelines due to its excellent capacity of suppressing hepatic gluconeogenesis and reducing blood glucose levels [9–11]. Besides, it was demonstrated as well that Metf possessed excellent treatment effect on some metabolic disorders such as polycystic ovary syndrome and gestational diabetes mellitus [12]. Camptothecin (CPT) represents a well-known anticancer agent and famous for the important poison of DNA topoisomerase I (TopoI) [13]. Underlying mechanisms of CPT and its derivatives inducing DNA damage were cleavable complex stabilization and its collision with the replication fork and transcription bubble [14,15]. Although Metf and CPT displayed excellent effect on their corresponding disease, the comprehensive therapeutic effect was seriously impaired by the limited drug delivery efficacy and off-target effects.

Polymeric materials-based nanoparticles (NPs) represent one of the most widely applied drug delivery systems in nanomedicine due to their excellent biocompatibility and high drug-loading efficacy [16–18]. Moreover, this type of NPs was easy to realize the structural modification such as decoration with tumor-homing peptides or aptamers on its surface for tumor targeting drug delivery [19–21]. However, large drug aggregates always formed during the process of NP self-assembly seriously lowers the drug loading capacity and encapsulation efficiency [22]. Besides, those extremely hydrophobic drugs such as CPT are always produce heterogeneous compositions and broad particle size distributions [23]. Therefore, developed a nano-platform with high drug-loading efficacy was one of most premises to achieve the satisfactory treatment effect.

In the present study, we aim to develop multifunctional nanoparticles with high drug-loading capacity for simultaneously improve the type 2 diabetes mellitus and treat malignant breast cancer. To realize the goal, the CPT was linked with the PLGA polymer *via* the pH-sensitive hydrazone bonds. By this was, the extremely hydrophobic CPT could be efficiently loaded by the PLGA nanoparticles and could be released under the acidic tumor microenvironment. For simultaneously loaded with Metf, the agents were physically loaded into the inner core of CPT-conjugated PLGA nanoparticles. Furthermore, CGKRK peptides were further decorated on the surface of the developed dual-drugs loaded nanoparticles to obtain the goal of precise tumor-targeting drug delivery. CGKRK peptide was designed to specially bind to heparan sulfate receptors highly expressed on a wide range of tumor cells [24,25]. By application of the developed tumor targetable and pH-sensitive polymer nanoparticles, we for simultaneously improve the T2DM and treat malignant breast cancer.

## Materials and methods

Methoxypoly (ethylene glycol) _3000_-poly (lactic-co-glycolic acid) _20000_-hydrazone bond-CPT (MPEG-PLGA-hyd-CPT) and maleimide-poly (ethylene glycol) _3400_-poly (lactic-co-glycolic acid) _20000_-hydrazone bond-CPT (Male-PEG-PLGA-hyd-CPT) were purchased from Jinan Daigang Biomaterial Co., Ltd (Shandong, China). The CGKRK peptide, abbreviated as C peptides, was synthesized by China Peptides Co., Ltd (Shanghai, China) while CPT and Metf were obtained from Xudong Haipu Pharmaceutical Co., Ltd (Shanghai, China). Cell Counting Kit-8 (CCK-8) and 0.25% Trypsin-EDTA were provided by Invitrogen (Carlsbad, CA, USA) and the annexin V-FITC apoptosis detection kit was purchased from BD Bioscience (San Diego, CA, USA). Dulbecco’s modified Eagle’s medium (DMEM) and 0.25% trypsin-EDTA were purchased by Gibco BRL (Carlsbad, CA, USA).

### Cell culture

The triple-negative breast cancer (TNBC) cell lines MDA-MB-231 and HCC1806 cells were achieved by the American Type Culture Collection (ATCC; Manassas, VA). For cell culture, the complete DMEM medium was prepared firstly and supplemented with 10% FBS, 100 U/mL penicillin and 100 μg/mL streptomycin. After that, the MDA-MB-231 cells and HCC1806 cells were cultured in the prepared DMEM medium.

### Development of animal models

Specific pathogen-free male BALB/c nude mice (six weeks old with weights of 20 ± 2 g) were provided by the JSJ Animal Ltd. (Shanghai, China). The mice with diabetes were firstly developed using the streptozotocin (STZ)-inducing method [26]. In brief, the streptozotocin was dissolved by sodium citrate (50 mM, pH 4.5). Then, the prepared solution was injected intravenously into mice at the dosage of 1.2 mg per 20 g body weight. After a week, the induction of diabetes was verified by measurement of fasting blood sugar. For establishment of tumor-bearing mice models, subcutaneous transplantation approach was applied due to multiple advantages such as easy to build, possess same tumor environment to the orthotopic ones, and less susceptible to infection [27]. In brief, 5 × 10^5^ MDA-MB-231 or HCC1806 cells were cultured in 100 µL PBS (pH 7.4). Then, the cell suspension was subcutaneously injected into the flanks of the developed diabetes mice. After the tumor volumes grown to 100 mm^3^ around, the animal models were subjected to animal experiments.

### Preparation of CNP/CPT-Metf

CNP/CPT-Metf was developed using the nanoprecipitation method [28]. In brief, 28 mg of MPEG-PLGA-hyd-CPT and 2 mg of Male-PEG-PLGA-hyd-CPT were dissolved in 1 mL of tetrahydrofuran (THF). After that, 10 mL of 0.2 M citrate buffer (pH 4.0) was supplemented and mixed thoroughly. Then, the solution was poured slowly into 10 mL of 0.2 M citrate buffer (pH 4.0) followed by gently stirring for 1 h. To remove the residual THF, the above solution was poured into a dialysis bag (MW cutoff, 14,000 Da) and dialyzed against 0.01 M PBS (pH 7.4) for 24 h. The purified solution was collected and poured into a penicillin bottle. For metformin loading, 4 mg metformin hydrochloride was supplemented and mixed for 24 h in the dark. Finally, the developed NP/CPT-Metf were achieved after centrifugation for 45 min at 12,000 rpm. For peptides modification, the obtained NP/CPT-Metf were resuspended with distilled water and poured into a penicillin bottle. Then, excess CGKRK peptide solution was added and incubated with nanoparticles for 6 h. After that, the peptides-functionalized nanoparticles were achieved by centrifugation for 45 min at 12,000 rpm. The NPs only loaded with CPT (NP/CPT) or metformin (NP/Metf) were developed using the same approach to above.

### Characterization of CNP/CPT-Metf

General physicochemical properties including particle sizes and zeta-potentials of the developed nanoparticle formulations were investigated by the Zetasizer Nano ZS90 (Malvern Instruments, Southborough, MA). For morphologies determination, all the developed nanoparticles were negative stained with sodium phosphotungstate solution followed by examination under the transmission electron microscope (TEM, JEM-1230, JEOL, Japan).

### In vitro *drug release assay*

The equilibrium dialysis method was applied to investigate the drug release behavior of CNP/CPT-Metf [25]. For experiments, 10 mg of CNP/CPT-Metf was dissolved by 1 mL PBS (pH 7.4) release medium supplemented with 0.1% Tween-80. Then the solution was transformed into a dialysis bag (MWCO = 8000 Da) followed by immersion in 50 mL of release medium. After that, the dialysis bag was incubated at 37°C with the shaking speed of 100 rpm. Then, a 0.2 mL of sample solution was obtained at pre-determined time points with an equal volume of fresh medium was immediately supplemented. Finally, the release of CPT or Metf was determined by HPLC analysis. Of great importance, drug release behavior of CNP/CPT-Metf was further investigated in pH 6.0 as well to evaluate the pH sensitivity in faintly acid environment.

### Cell uptake and intracellular drug distribution

Cell uptake of CNP/CPT-Metf was performed on the TNBC cell lines (MDA-MB-231 and HCC1806 cells) and compared with NP/CPT-Metf. For visualization of nanoparticles, both of CNP/CPT-Metf and NP/CPT-Metf were labeled with FITC. 1 × 10^4^ MDA-MB-231 cells or HCC1806 cells were seeded on each well of 24-well plates. After grown for an overnight, the old medium in each well was removed and supplemented with 1-mL fresh medium containing different concentrations of FITC-CNP/CPT-Metf or FITC-NP/CPT-Metf. Then, the cells were allowed to interact with nanoparticles for one hour. Subsequently, the nanoparticle solution in each well was removed and cells were washed twice by cold PBS. For qualitative analysis, the cells were fixed with 4% paraformaldehyde solution and then analyzed under a fluorescent microscopy (Leica DMI4000 B, Germany). For quantitative analysis, the cells were incubated with 0.25% Trypsin-EDTA for 1 min. Then, the cell pellets were collected by centrifugation at 1000 g followed by analysis using the flow cytometry system (BD, USA).

### Anti-proliferation assay

Anti-proliferation activity of CNP/CPT-Metf was determined by the CCK-8 assay [29]. For experiments, MDA-MB-231 cells or HCC1806 cells were seeded in 96-well plates at the density of 5 × 10^3^ per well. After the cells were grown for 90% confluence, the fresh medium containing 100 µg/mL of NP/CPT, NP/Metf, NP/CPT-Metf, and CNP/CPT-Metf, respectively, was added into each well. Then, the cells were respectively incubated with nanoparticles for 12 h, 24, 36, and 48 h. At each time points, the cells were carefully washed twice with cold PBS to remove the unattached cells and residual drugs. After that, each well 100 µL fresh FBS free culture medium was added into each well and interacted with 10 µL CCK8 for 1 h. Finally, the absorbancy of each well was analyzed *via* a microplate reader.

### In vitro *cytotoxicity*

Cytotoxicity of CNP/CPT-Metf to MDA-MB-231 cell or HCC1806 cells was evaluated using the Annexin V-FITC/PI double staining method [24]. 1 × 10^5^ DA-MB-231 or HCC1806 cells were seeded on each well of six-well plates and cultured for an overnight. Then, the old medium in each well was removed and supplemented with fresh medium containing 100 µg/mL of NP/CPT, NP/Metf, NP/CPT-Metf, and CNP/CPT-Metf, respectively. After 24 h incubation, the nanoparticle solution in each well was removed and treated with 0.25% trypsin-EDTA for 1 min. Then, the cell pellets were collected by centrifugation at 1000 g followed by staining with Annexin V-FITC/PI and analyzed by the flow cytometry system (BD, USA).

### *Tumor targeting assay* in vivo

After the tumor volumes grown to 100 mm^3^ around, the animal models were randomly divided into two groups (*n = 6*) and treated with NP/CPT-Metf and CNP/CPT-Metf, respectively. After 24 h, all the mice were euthanatized with primary organs (heart, liver, spleen, lung, kidney, and tumor) were achieved. Then, the achieved organs were washed three times by PBS (pH 7.4) followed by homogenate for nanoparticle concentration analysis using the HPLC system.

### Treatment of mice models

After the tumor volumes grown to 100 mm^3^ around, the animal models were randomly grouped (*n = 10*) and respectively treated by saline, NP/CPT, NP/Metf, NP/CPT-Metf and CNP/CPT-Metf. Then, the tumor growth rate was observed every two days by recorded the tumor volume changes. Moreover, the medium survival time of each group was recorded and analyzed by the Kaplan-Meier survival curve. The effect of CNP/CPT-Metf on the alleviation of T2DM was investigated by monitoring the changes of fasting blood glucose levels (FBGL) and oral glucose tolerance test (OGTT) of the diabetic mice.

### Statistical analysis

The comparison between two groups were performed by the unpaired student’s t test while multiple-group analysis was performed by the one-way ANOVA with Bonferroni tests. The statistical analysis here was performed by GraphPad Prism 5.0 with p < 0.05 was defined as statistical significance.

## Results

**Figure 1.**
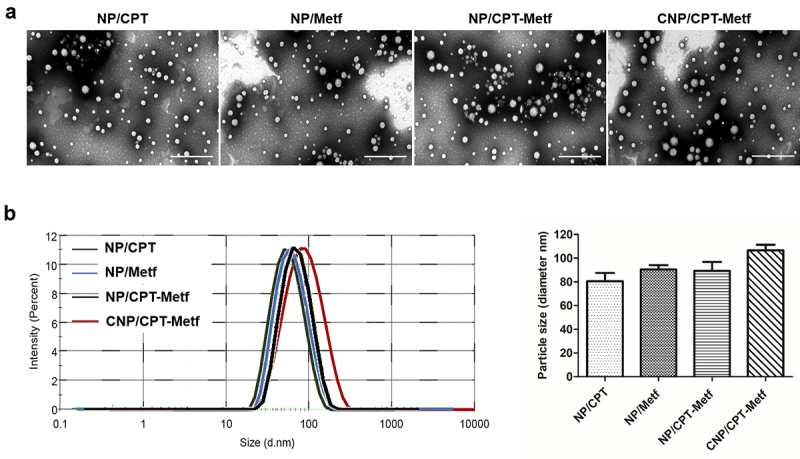
Characterization of nanoparticles. (a) TEM images of NP/CPT, NP/Metf, NP/CPT-Metf, and CNP/CPT-Metf. The bar represented as 1 μm. (b) Medium particle sizes of NP/CPT, NP/Metf, NP/CPT-Metf, and CNP/CPT-Metf determined by DLS analysis.

As demonstrated in Figure 1a, all the developed nanoparticles displayed a near spherical morphology. Besides, the nanoparticles have a uniform shape and negligible aggregation indicated excellent dispersibility. Results shown that the diameter of CNP/CPT-Metf was 96.58 ± 1.89 nm with other nanoparticles including NP/CPT, NP/Metf, and NP/CPT-Metf were 80.57 ± 1.23 nm, 90.49 ± 1.65 nm, and 88.27 ± 1.08 nm, respectively (Figure 1b). Moreover, zeta potentials of various nanoparticles were determined as well. The values for each nanoparticle formulation were −23.22 ± 2.21 mV (NP/CPT), −20.17 ± 1.06 mV (NP/Metf), −18.83 ± 1.32 mV (NP/CPT-Metf), and −16.36 ± 1.59 mV (CNP/CPT-Metf), respectively.

### Drug release behavior

**Figure 2.**
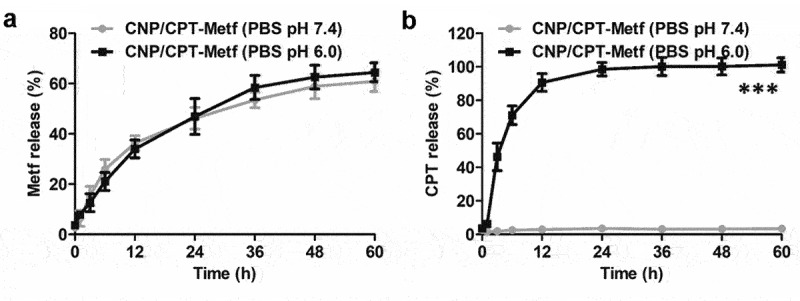
Drug release behavior of CNP/CPT-Metf in different medium investigated by the dialysis experiments. Release of Metf (a) and CPT (b) from the CNP/CPT-Metf in different release medium. The PBS with pH of 6.0 was represented as the pH in tumor microenvironment while the pH of 7.4 was represented as the pH in physiologic condition. ***P < 0.001 significantly different from the group of CNP/CPT-Metf (PBS pH 7.4).

Drug release behavior was investigated in the medium of PBS with different pH conditions. The PBS with pH of 6.0 was represented as the pH in tumor microenvironment while the pH of 7.4 was represented as the pH in physiologic condition. As shown in Figure 2a, controlled release behavior was observed for Metf in both pH 6.0 and 7.0. Additionally, similar results of cumulative Metf release was obtained at 60 h in the medium of pH 6.0 and 7.4 with the values of 65.84% ± 2.17% (pH 6.0) and 63.51 ± 1.83% (pH 7.4). These results indicated that the release of Metf from CNP/CPT-Metf was not affected by pH conditions. However, investigation of CPT release in pH 6.0 revealed a significant different behavior from that in pH 7.4. As demonstrated in Figure 2b, a burst release behavior was achieved for CPT in pH 6.0 with a cumulative CPT release of 90.92 ± 0.43% at 12 h. In contrast, there was almost negligible CPT signal was detected in the medium of pH 7.4 with a cumulative CPT release of 2.12% ± 0.43% at 60 h. These results indicated that extremely low CPT was released from CNP/CPT-Metf in the PBS with pH of 7.4.

### Cell uptake assay

**Figure 3.**
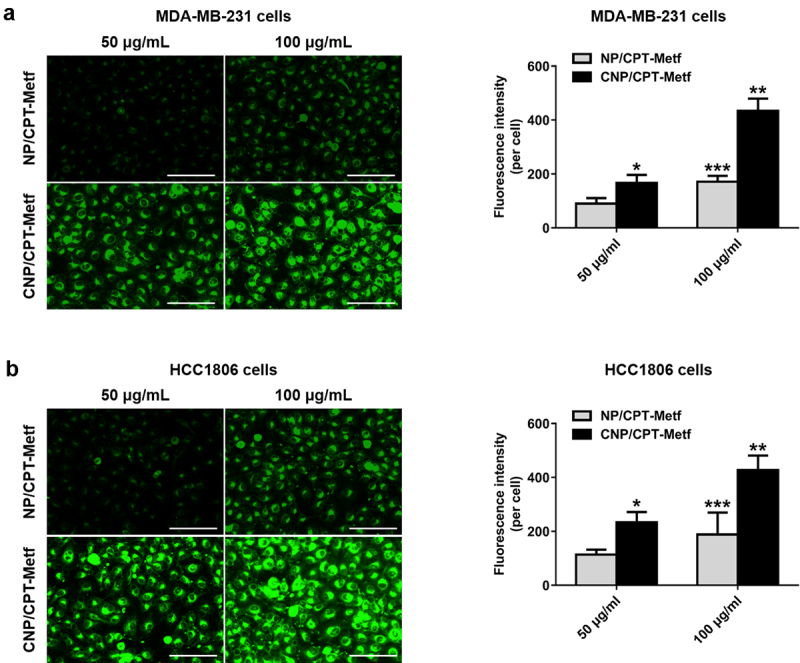
Cellular uptake of FITC-labeled unmodified NP/CPT-Metf and CNP/CPT-Metf on MDA-MB-231 and HCC1806 cells. (a) Qualitative and quantitative analysis of cell uptake of CNP/CPT-Metf and compared with the NP/CPT-Metf on MDA-MB-231 cells. The bar represents 100 μm. (c) Qualitative and quantitative analysis of cell uptake of CNP/CPT-Metf and compared with the NP/CPT-Metf on HCC1806 cells. The bar represents 100 μm. *P < 0.05, **P < 0.01, and ***P < 0.001 significantly different from the NP/CPT-Metf group.

To verify tumor targeting ability of the developed CNP/CPT-Metf, cell uptake assay was respectively performed on the MDA-MB-231 and HCC1806 cells. As shown in Figure 3(a,b), the cells treated by CNP/CPT-Metf exhibited significant stronger fluorescent intensity than the cells treated by NP/CPT-Metf at different nanoparticle concentrations. Such results were further confirmed by the quantitative analysis results since signal higher amounts of nanoparticles was accumulated in the cells treated by CNP/CPT-Metf than CNP/CPT-Metf. These results together confirmed that modification of CGKRK peptides was favorable for cell uptake of drugs.

### *Anti-tumor effect of CNP/CPT-Metf* in vitro

**Figure 4.**
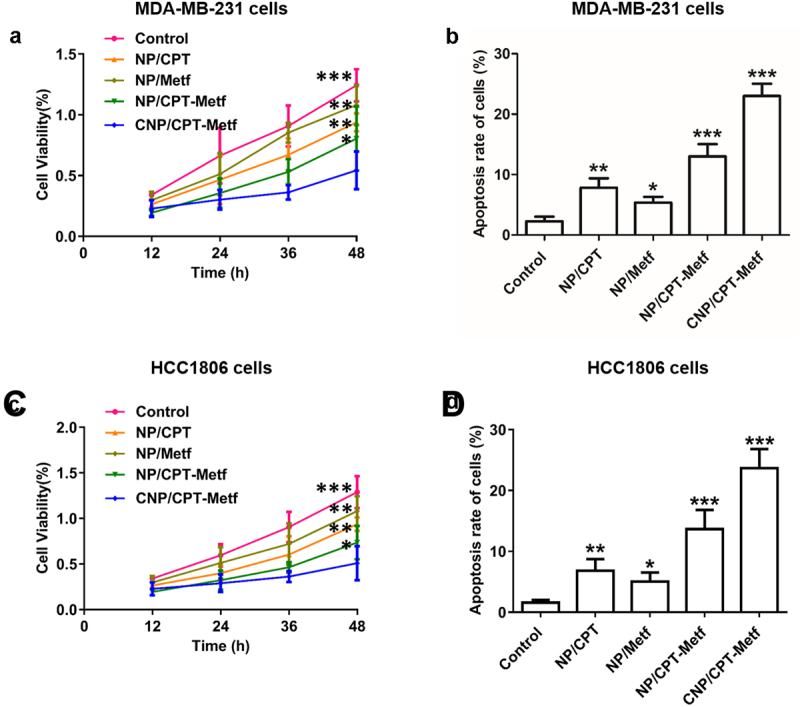
Evaluation of anti-tumor effect of CNP/CPT-Metf *in vitro*. (a) Growth of MDA-MB-231 cell lines after treated by different nanoparticle formulations determined by CCK-8 assay. (b) Apoptosis rate of MDA-MB-231 cell lines after treated by different nanoparticle formulations investigated by the Annexin V-FITC/PI double staining method. (c) Growth of HCC1806 cells after treated by different nanoparticle formulations determined by CCK-8 assay. (d) Apoptosis rate of HCC1806 cells after treated by different nanoparticle formulations investigated by the Annexin V-FITC/PI double-staining method. *P < 0.05, **P < 0.01, and ***P < 0.001 significantly different from the control group.

Given the excellent breast cancer cells targeting ability of CNP/CPT-Metf, there were logical reasons to assume that the CNP/CPT-Metf possess strong capacity of anti-proliferation and cell apoptosis inducing. Anti-proliferation ability of CNP/CPT-Metf on MDA-MB-231 cells and HCC1806 cells were investigated by CCK-8 assay. As demonstrated in Figure 4(a,c), significant higher toxicity was obtained in the cells treated by CNP/CPT-Metf when compared with NP/CPT-Metf. For other groups, NP/CPT exhibited a relative higher toxicity on MDA-MB-231 cells (Figure 4a) and HCC1806 cells (Figure 4c) than NP/Metf, indicated a superior anti-tumor effect of CPT to Metf.

Further quantitative flow cytometry analysis was performed to investigate the ability of CNP/CPT-Metf to induce cell apoptosis and compared with NP/CPT-Metf. As shown in 4B the cell apoptosis rate of CNP/CPT-Metf treated MDA-MB-231 cells was 29.18 ± 2.71% which was dramatically higher than the MDA-MB-231 cells treated by NP/CPT-Metf with a value of 15.23 ± 2.14%. For the MDA-MB-231 cells treated by NP/CPT and NP/Metf, the percentage of cell apoptosis rate were 9.23 ± 1.07% and 6.15 ± 1.39%, respectively. Similar results were obtained for the HCC1806 cells. As shown in Figure 4d, the cell apoptosis rate of CNP/CPT-Metf treated HCC1806 cells was 26.27 ± 1.74% which was dramatically higher than the HCC1806 cells treated by NP/CPT-Metf with a value of 13.73 ± 2.04%. For the HCC1806 cells treated by NP/CPT and NP/Metf, the percentage of cell apoptosis rate were 6.21 ± 1.33% and 5.36 ± 2.37%, respectively.

### *Tumor targeting assay* in vivo

**Figure 5.**
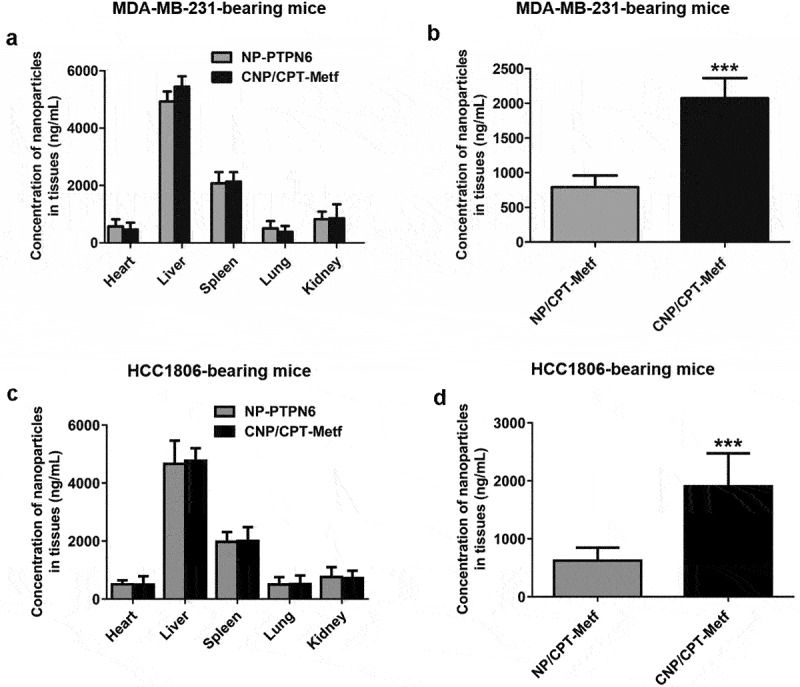
Biodistribution of nanoparticles *in vivo*. (a) Quantitative analysis of the nanoparticle concentration in different organs including heart, liver, spleen, lung, and kidney from the MDA-MB-231-bearing mice. (b) Quantitative analysis of the nanoparticle concentration in tumor tissues from the MDA-MB-231-bearing mice. (c) Quantitative analysis of the nanoparticle concentration in different organs including heart, liver, spleen, lung, and kidney from the HCC1806-bearing mice. (d) Quantitative analysis of the nanoparticle concentration in tumor tissues from the HCC1806-bearing mice. ***P < 0.001 significantly different from the NP/CPT-Metf group.

To evaluate the drug delivery efficacy of CNP/CPT-Metf *in vivo*, animal models bearing with Type 2 Diabetes Mellitus and breast cancer were applied. As shown in Figure 5(a–d), similar bio-distribution of nanoparticles was observed between the mice treated by CNP/CPT-Metf and NP/CPT-Metf. However, it was further demonstrated that significant lower accumulation of NP/CPT-Metf than CNP/CPT-Metf was detected in the breast cancer tissues.

### *Anti-tumor effect of CNP/CPT-Metf* in vivo

**Figure 6.**
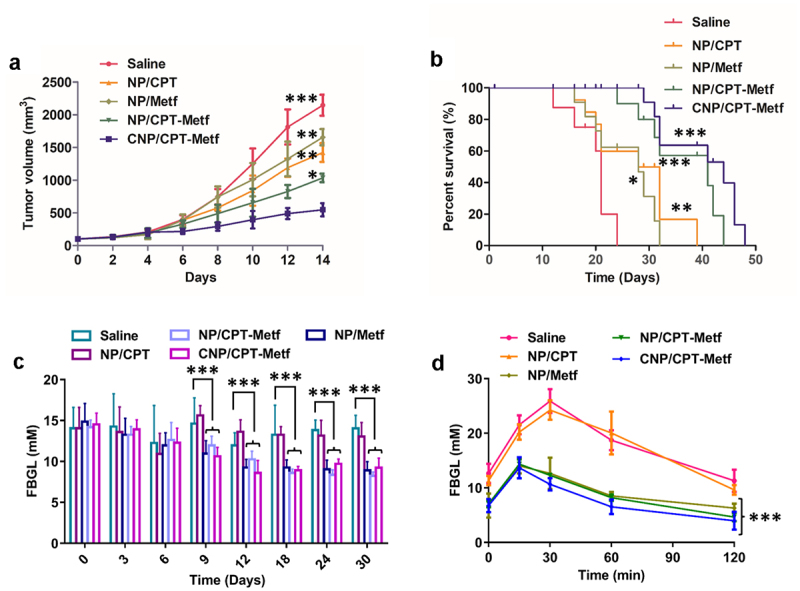
Evaluation of therapy effect of CNP/CPT-Metf on mice models. (a) Change in tumor volumes of the mice models during the 14-day experimental period. (b) Kaplan-Meier survival curve of mice models treated with different nanoparticle formulations. (c) The FBGL of mice models during the drug treatment. (d) Changes of OGTT was measured after drug administration. *P < 0.05, **P < 0.01, and ***P < 0.001 significantly different from the control group (saline group).

Results shown that CNP/CPT-Metf treated mice displayed the lowest tumor progress rate and tumor weights among all groups. (Figure 6(a,b)). For other groups, the NP/CPT-Metf treated mice exhibited obvious lower tumor growth rate and tumor weights than the NP/CPT or NP/Metf treated mice, suggested an excellent combination therapy effect of CPT and Metf. Moreover, a relative stronger anti-tumor effect was observed for NP/CPT than NP/Metf which was similar to the cellular experimental results.

### CNP/CPT-Metf significantly alleviated the Type 2 Diabetes Mellitus in diabetic mice

As shown in Figure 6c, the FBGL of diabetic mice models was dramatically higher than the normal control mice. However, after daily administration of various nanoparticle formulations, the FBGL of diabetic mice was dropped to a varying degree in different treatments. The mice received the treatment of CNP/CPT-Metf and NP/CPT-Metf obtained the most excellent therapy effect, which was basically reduced to the normal level after treatment. Additionally, the oral glucose tolerance test of the diabetic mice was further evaluated after received different treatments. As shown in Figure 6d, the mice without any treatments but saline had significantly higher FBGL than other groups during the 120 min OGTT, indicated that the diabetic mice have a seriously impaired glucose tolerance. Importantly, after the mice was treated by CNP/CPT-Metf and NP/CPT-Metf, the glucose tolerance was obviously recovered and the effect was dramatically superior to the treatment of NP/CPT or NP/Metf. Taking these results together, the CNP/CPT-Metf was demonstrated to be able of significantly alleviating the type 2 diabetes mellitus in diabetic mice.

## Discussion

Previous studies have demonstrated that diabetes was one of the most important risk factors for developing breast cancer in women [30,31]. More importantly, patients with breast cancer and diabetes have significant higher risk for all-cause mortality than those without diabetes [5,32]. However, the treatment strategies for patients with breast cancer and diabetes are still not efficient enough. In the present study, we aim to develop a novel strategy for efficiently deliver therapeutic agents to the disease sites for active targeting therapy. To achieve that goals, multifunctional nanoparticles co-loaded with CPT and Metf were developed by PLGA co-polymer. Importantly, the developed CNP/CPT-Metf was characterized by tumor targetable and pH-sensitive. It was because of on the the surface of NP/CPT-Metf was modified with tumor-homing peptides and the CPT was conjugated with PLGA polymer by pH-sensitive hydrazone bonds.

Design of a befitting drug delivery system is critical for tumor targeting treatment [33]. Nanoparticles made with biodegradable polymer represents the most promising carriers of therapeutic agents because of their multiple advantages over conventional drug delivery vehicles [34,35]. Among those polymers, PLGA have been widely used in the field of drug deliver because of its low toxicity and excellent biocompatibilities [36–38]. Therefore, in the present study, the PLGA was selected as carrier material for development of dual-drugs loaded nanoparticles. The nanoparticles developed here showed an uniform shape and negligible aggregation. Besides, 100 nm around particle size was achieved by CNP/CPT-Metf. As demonstrated previously, particles with average size of 100 nm tend to represents the most optimal range for leveraging the EPR effect [39,40].

Generally, the tumor microenvironment was characterized by mildly acidity due to acidic metabolic waste products accumulation [41]. Based on this, multiple acid-sensitive nanoparticles were designed for smart drug delivery [42,43]. These smart drug delivery systems were characterized by not only able of efficiently deliver drugs to targeting sites but also low toxicity to normal tissues [41,43]. In the present study, the CPT was conjugated with PLGA polymer hydrazone bonds, which was highly sensitive to the weak acidic tumor microenvironment. By this way, the structure of CPT-PLGA can be remain integrity and completely released once they were exposed to the weak acidic tumor microenvironment. Drug release behavior investigation demonstrated that the developed CNP/CPT-Metf was stable enough when incubated in neutral release medium while extremely instability in the weak acid medium.

CGKRK is a well-known tumor targeting peptides discovered by phage display [24,25]. A wide range of studies have used CGKRK for active tumor targeting drug delivery due to its high affinity to heparan sulfateea sulfated polysaccharide which was highly expressed on multiple tumor cells [24,25]. In the present study, the CGKRK peptides were decorated on the surface of dual-drugs loaded nanoparticles for realizing active tumor targeting drug delivery. Cellular experiments demonstrated that modification of CGKRK peptides was favorable for cell uptake of drugs with stronger signal was observed in the CNP/CPT-Metf-treated cells than the NP/CPT-Metf-treated cells. Importantly, the excellent breast cancer cells targeting ability of CNP/CPT-Metf, resulted in strong capacity of anti-proliferation and cell apoptosis inducing. It was showed that significant higher cytotoxicity was achieved in the cells treated by CNP/CPT-Metf when compared with NP/CPT-Metf.

Multiple factors such as the reticuloendothelial system, enlarged endothelial gaps in tumors, and dense stroma in the tumor microenvironment affect the efficient drug delivery to tumor tissues by nanoparticles [44,45]. An ideal drug delivery system should be efficiently delivery of drugs to disease sites and reduce the drug distribution at unwanted tissues as few as possible. The present study was aimed to developed a tumor targetable and pH-sensitive nanoparticle for efficiently drug deliver. As demonstrated that the prepared CNP/CPT-Metf mediated more accumulation of drugs at tumor sites than the unmodified NP/CPT-Metf. Additionally, high tumor affinity of CNP/CPT-Metf resulted in stronger anti-tumor effect than NP/CPT-Metf. Interestingly, it was observed that CNP/CPT-Metf significantly alleviated the type 2 diabetes mellitus in diabetic mice by efficiently recover the glucose tolerance.

### Conclusion

In the present study, multifunctional nanoparticles co-loaded with CPT and Metf were developed based on PLGA polymers for simultaneously improve the type 2 diabetes mellitus and treat malignant breast cancer. The novel nanoparticles were characterized by tumor targetable and pH-sensitive. The anti-tumor drugs CPT was conjugated with PLGA *via* the pH-sensitive hydrazone bonds and then formed to CPT-conjugated PLGA nanoparticles by nanoprecipitation method. Subsequently, the classical antidiabetic agent Metf was physically loaded into the inner core of CPT-conjugated PLGA nanoparticles. To achieve the goal of tumor targeting, the namely tumor homing CGKRK peptides was further decorated on the surface of NP/CPT-Metf to target delivery of drugs to tumor tissues. The developed nanoparticles showed a sufficient sensitivity to the weak acidic tumor microenvironment as demonstrated by *in vivo* drug release assay. After modification with CGKRK peptides, accumulation of drugs in cells and tumor tissues was significantly enhanced, which further resulted in a reinforced anti-tumor effect both *in vitro* and *in vivo*. Additionally, it was further demonstrated that CNP/CPT-Metf was able of significantly alleviating the Type 2 Diabetes Mellitus in diabetic mice.

## Data Availability

The data are available from the authors upon request.
